# Radiofrequency Pulmonary Vein Isolation without Esophageal Temperature Monitoring: Contact-Force Characteristics and Incidence of Esophageal Thermal Damage

**DOI:** 10.3390/jcm11236917

**Published:** 2022-11-23

**Authors:** Stefan Hartl, Carsten auf der Heiden, Alexandru Bejinariu, Lukas Clasen, Anna Füting, Stephan vom Dahl, Tom Lüdde, Malte Kelm, Hisaki Makimoto

**Affiliations:** 1 Department of Cardiology, Pulmonary Diseases and Vascular Medicine, Medical Faculty, Heinrich-Heine University, 40225 Düsseldorf, Germany; 2 Department of Electrophysiology, Alfried Krupp Hospital, 45131 Essen, Germany; 3 Department of Medicine, Witten/Herdecke University, 58455 Witten, Germany; 4 Department of Cardiology, Rhythmology and Angiology, Josephs-Hospital Warendorf, Academic Teaching Hospital, University of Münster, 48231 Warendorf, Germany; 5 Department of Gastroenterology, Hepatology and Infectious Diseases, Medical Faculty, Heinrich-Heine University, 40225 Düsseldorf, Germany; 6 CARID, Cardiovascular Research Institute Düsseldorf, Medical Faculty, Heinrich-Heine-University, 40225 Düsseldorf, Germany

**Keywords:** atrial fibrillation, contact force, ablation, pulmonary vein isolation, esophageal thermal lesion

## Abstract

Esophageal thermal lesions following pulmonary vein isolation (PVI) for atrial fibrillation (AF) potentially harbor lethal complications. Radiofrequency (RF)-PVI using contact force-technology can reduce collateral damage. We evaluated the incidence of endoscopically detected esophageal lesions (EDEL) and the contribution of contact force to esophageal lesion formation without esophageal temperature monitoring. One hundred and thirty-one AF patients underwent contact force-guided RF-PVI. Contact force, energy, force-time-integral, and force-power-time-integral were adopted. During PVI at the posterior segment of the wide antral circumferential line, limits were set for energy (30 W), duration (30 s) and contact force (40 g). Ablations were analyzed postero-superior and -inferior around PVs. Endoscopy within 120 h identified EDEL in six patients (4.6%). In EDEL(+), obesity was less frequent (17% vs. 68%, *p* = 0.018), creatinine was higher (1.55 ± 1.18 vs. 1.07 ± 0.42 mg/dL, *p* = 0.016), and exclusively at the left postero-inferior site, force-time-integral and force-power-time-integral were greater (2973 ± 3267 vs. 1757 ± 1262 g·s, *p* = 0.042 and 83,547 ± 105,940 vs. 43,556 ± 35,255 g·J, *p* = 0.022, respectively) as compared to EDEL(−) patients. No major complications occurred. At 12 months, arrhythmia-free survival was 74%. The incidence of EDEL was low after contact force-guided RF-PVI. Implementing combined contact force-indices on the postero-inferior site of left-sided PVs may reduce EDEL.

## 1. Introduction

Catheter ablation for pulmonary vein isolation (PVI) is the cornerstone in the interventional treatment of symptomatic atrial fibrillation (AF) [1]. Independently of the energy source applied, the procedure is safe and severe complications are rare [2,3,4]. One of these rare, but in the majority of cases, fatal complications is the formation of an atrio-esophageal fistula (AEF), which typically develops within weeks after ablation based on an esophageal thermal injury. Deeper and transmural esophageal lesions are more susceptible to progress into an AEF [5]. The incidences of endoscopically detected esophageal lesions (EDEL) often exceeded 10% [5,6,7] and were reported in up to 47% [8] in previous studies. The formation of esophageal thermal lesions depends on many factors and in radiofrequency (RF) ablation, particularly on the applied energy, ablation duration and contact force [6]. In contrast, these parameters alone or as combined contact force indices are also determinants for the creation of effective ablation lesions [9,10,11], which requires a careful risk-benefit balance regarding a successful ablation without compromising safety. Different strategies have been investigated to prevent esophageal complications (e.g., esophageal deviation, esophageal cooling) but have not been established due to limited or less convincing data [12,13]. The benefit of esophageal temperature monitoring during the ablation procedure is still under debate. Currently, it has a class IIa/C recommendation in the guidelines [14]. The recent, randomized OPERA trial did not show advantages of esophageal temperature monitoring relating to the occurrence of esophageal lesions in RF ablation [15]. Also, in other studies, esophageal lesions were reported despite esophageal temperature monitoring [5,7,16,17]. The conflicting findings regarding esophageal temperature monitoring during PVI necessitate additional data and lead the focus on the development of further preventive strategies, such as the identification of procedure- or patient-specific factors that are associated with esophageal lesion formation. Meanwhile, contact force-sensing catheters have been proven to efficiently guide point-by-point RF ablation. The underlying technology could reduce collateral tissue damage by avoiding high contact force during PVI at the posterior segment around the PV which is located in anatomic proximity to the esophagus. So far, little is known about the contribution of applied contact force to esophageal thermal lesion formation, particularly with respect to combined contact force indices which display multipliers of contact force, time and energy.

The aims of this single-center study were to evaluate (1) the incidence of EDEL in clinical practice without esophageal temperature monitoring during contact force-guided RF-PVI and (2) the potential contribution of applied contact force to the formation of EDEL. As a secondary outcome, freedom from atrial arrhythmia was assessed.

## 2. Materials and Methods

### 2.1. Study Population

**Figure 1:**
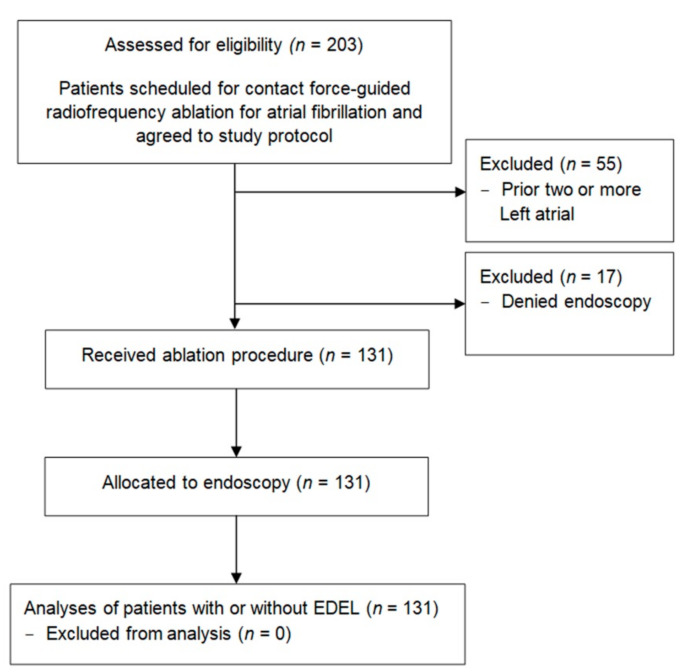
Patient enrollment. CF indicates contact force; RF, radiofrequency; LAT, left atrial tachycardia; LA, left atrial and EDEL, endoscopically detected esophageal lesion.

Consecutive patients who were scheduled for contact force-guided RF ablation of AF as an index or re-do procedure were assessed for eligibility in this single-center prospective study. Inclusion criteria were symptomatic paroxysmal or persistent AF with or without left atrial tachycardia (LAT), patient preference and ineffectiveness, and contraindications or refusal of antiarrhythmic drugs according to the guidelines [18,19]. Patients who received re-do procedures were only included if wide antral circumferential PV re-ablation (WACA) was performed. Patients who agreed to undergo postprocedural endoscopy and agreed to the study protocol were enrolled in the final analyses (Figure 1). Exclusion criteria were intracardiac thrombi, known severe esophageal disease and re-do procedures with conventional gap ablation. Patients with two or more prior left atrial ablation procedures were also excluded.

### 2.2. Ablation Procedure Protocol

**Figure 2:**
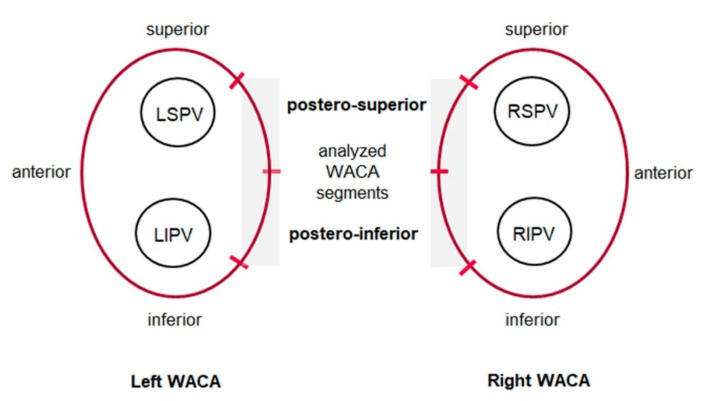
Schematic depiction of the investigated ablation sites at the posterior segments of the wide antral circumferential ablation line around the pulmonary veins. Contact force parameters were analyzed at four pre-defined sites (shaded in grey) of the WACA line (red) that are in close anatomic proximity to the esophagus: the postero-superior and postero-inferior segments around the left and right pulmonary veins. LSPV indicates left superior pulmonary vein; LIPV, left inferior PV; RSPV, right superior PV; RIPV, right inferior PV; and WACA, wide antral circumferential ablation.

A detailed summary of our standardized RF ablation procedure has been described previously [20]. Intracardiac thrombi were ruled out by transesophageal echocardiography. Contact force-guided RF-PVI was performed under conscious sedation by an experienced electrophysiologist. After the femoral venous puncture, a 6F catheter was advanced and placed in the coronary sinus. Two SL1 sheaths (St. Jude Medical/Abbott, Plymouth, MN, USA) were introduced into the LA following a double transseptal puncture under fluoroscopic and/or echocardiographic guidance. Intravenous heparin was administered as needed to achieve an activated clotting time > 300 s throughout the entire procedure. Selective PV angiography was performed to visualize the PV ostia. To achieve PVI, a wide antral circumferential ablation line was applied around the left- and right-sided PVs by means of contact force-guided point-by-point RF ablation using an irrigated quadripolar ablation catheter with a 3.5 mm tip (ThermoCool^®^ SmartTouch™, Biosense Webster, Inc., Irvine, CA, USA) under the guidance of a 3D mapping system (CARTO, Biosense Webster, Inc. Irvine, CA, USA). During PVI at the posterior segment of the wide antral line, an upper limit was set for energy (30 W; 17 mL/min flow), ablation duration (30 s) and contact force (40 g). With respect to the anatomical proximity to the esophagus, four predefined posterior LA segments of the wide antral circumferential ablation line were subject to further investigation of the applied contact force: right postero-superior, right postero-inferior, left postero-superior and left postero-inferior, respectively (Figure 2). Values for ablation at the anterior segment were set to a maximum of 40 W, 30 mL/min flow based on previous studies [21,22]. A fluoroscopy integrating system was implemented to reduce radiation exposure [23]. A multipolar diagnostic catheter (LASSO^®^, Biosense Webster, Inc., Irvine, CA, USA) was used to map and demonstrate PVI. The procedures were performed without esophageal temperature monitoring. PVI was the procedural endpoint and was confirmed by documenting a bidirectional block (entrance and exit block) at least 20 min after the last ablation. No additional ablations beyond PVI were applied and no posterior wall isolation was performed. In cases of left atrial tachycardias, further ablation was performed at the operator’s discretion. A figure of eight suture was applied during sheath removal to achieve hemostasis at the venous access site, and finally, a pressure bandage was put on. Echocardiography was performed to rule out pericardial effusion. All patients were recommended to take proton pump inhibitors for 6 weeks after the ablation.

### 2.3. Post Procedure Observation

The patients were observed on the cardiovascular ward with monitoring for at least 12 h and were discharged from our institute on the day after the procedure. Complications were documented as procedure-specific or procedure-related adverse outcomes, whereby major complications were defined as a permanent injury or death, the necessity of any interventional treatment or a prolonged hospitalization [24]. Asymptomatic EDEL were separately listed and not included in the overall complication rate.

### 2.4. Assessment of Contact-Force Parameters

The following parameters were extracted from the CARTO system (Biosense Webster, Inc. Irvine, CA, USA) for further analyses: contact-force (g), energy (J) and ablation duration(s). The force-time-integral (g·s) and the force-power-time-integral (g·J) were calculated according to the following formulas:

$$\mathrm{Force}\text{-}\mathrm{time}\text{-}\mathrm{integral}=\int \mathrm{Force}\left(g\right)\mathrm{dt}$$

$$\mathrm{Force}\text{-}\mathrm{power}\text{-}\mathrm{time}\text{-}\mathrm{integral}=\int \left\{\mathrm{Force}\left(g\right)\right\}*\left\{\mathrm{Power}\left(W\right)\right.\}\;\mathrm{dt}$$

Contact-force parameters were assessed for ablation applications at the posterior segments of the WACA line, e.g., postero-superior and postero-inferior around the left- and right-sided PVs (Figure 2).

### 2.5. Esophagogastroduodenoscopy and Classification of Endoscopically Detected Esophageal Lesions

All patients were scheduled for endoscopy (Olympus, Tokyo, Japan) under conscious sedation within 120 h after the ablation procedure while being continuously monitored. The findings were documented, reviewed and confirmed by two experienced gastroenterologists independently from each other. EDEL was assigned to typical mucosal defects in regions with close proximity to the LA posterior wall. The anatomical characterization of EDEL was based on the Kansas City classification (KCC). KCC type 1: erythema without disruption of the mucosa. Type 2a: superficial ulcers involving the mucosa only. Type 2b: deeper ulceration involving the muscularis externa. Type 3a: esophageal perforation without AEF. Type 3b: esophageal perforation and atrio-esophageal fistula [5].

### 2.6. Follow-Up

Patients with EDEL were urged to report potential symptoms and at least one follow-up endoscopy was recommended after 1–2 weeks to document the course of lesion formation. Routine rhythm follow up took place in the university outpatient clinic at 3 and 12 months including 3–7 day Holter monitoring, or more frequently in case of AF symptoms. Documented AF recurrences of at least 30 s after a 3 month blanking period were calculated as failure. Consultations with referring centers and telephonic interviews with patients were processed to complete follow up.

### 2.7. Statistical Analysis

Continuous data are given as mean with standard deviation (SD) or the median with interquartile range and were compared using the Student’s *t*-test. Categorical data are shown as numbers and percentages and were compared using the chi-square or the Fisher’s exact test, respectively. Event-free survival was estimated using the Kaplan-Meier method. A *p*-value < 0.05 was considered statistically significant. Data processing and analysis were performed using SPSS (IBM SPSS Statistics, Version 22.0, Armonk, NY, USA).

## 3. Results

Baseline characteristics are summarized in Table 1. In total, 131 patients with symptomatic AF underwent contact force-guided RF ablation and were followed up by endoscopy within 120 h (Figure 1). Mean patient age was 65.5 ± 10.3 years, 57% of patients were male and the mean CHA_2_DS_2_-VASc score was 2.5 ± 1.6. All targeted PVs were successfully isolated. Five patients (3.8%) presented with additional left atrial tachycardias and required further ablation: three left atrial macroreentrant tachycardias (mitral isthmus or anterior line ablation were performed, respectively), 2 PV tachycardias (PVI only) and 1 focal tachycardia originating from the left atrial appendage (focal ablation). The total procedure time was 134 ± 30 min. All procedural data are summarized in Table 2 and Table 3. Rhythm follow-up was available for 119 patients (91%). At a median follow-up time of 12 months (IQR 9;18), the estimated probability of arrhythmia-free survival was 74% for patients with paroxysmal and persistent AF.

### 3.1. Esophageal Endoscopic Findings and Periprocedural Adverse Events

**Figure 3:**
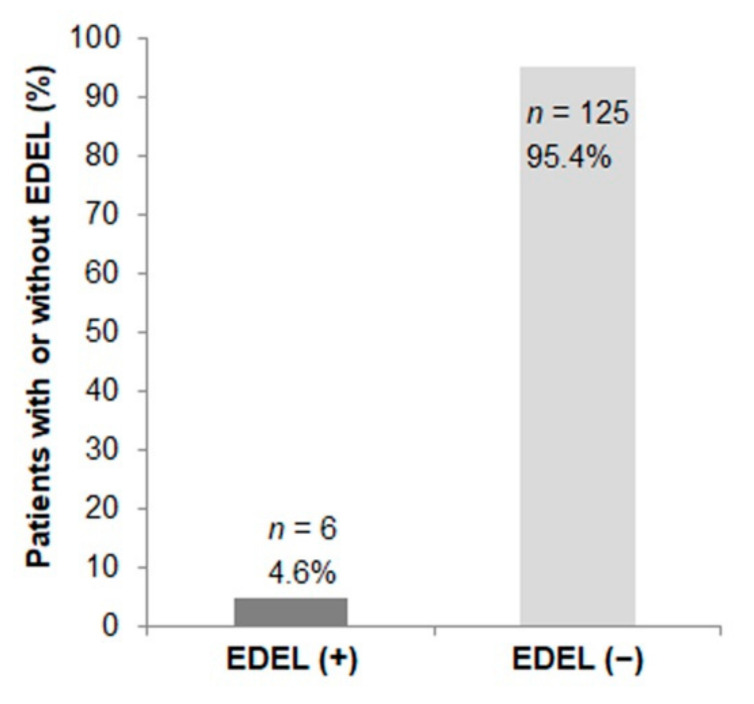
Incidence of endoscopically detected esophageal lesions (EDEL). Patients with EDEL were all symptom-free. According to the Kansas City classification (KCC) [5], one type 1 lesion (erosion) and five type 2a lesions (superficial ulceration) were detected. No deep ulceration, esophageal perforation or atrio-esophageal fistula was observed.

**Figure 4:**
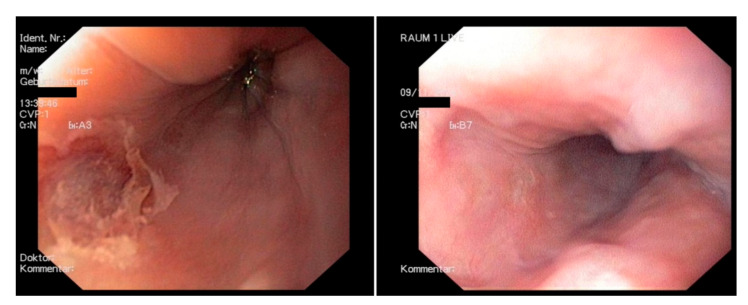
Representative image of an endoscopically detected esophageal lesion (EDEL) early and five weeks after contact force RF-PVI. Left: Endoscopy documents a superficial mucosal ulceration early after ablation, 40 × 20 mm at 30 cm from the incisors (Kansas City classification type 2a), which was asymptomatic to the patient. Right: Healing with residual scarring 5 weeks after ablation.

Esophageal thermal lesions were diagnosed in 6 of 131 patients (4.6%) (Figure 3) whereby five were classified as KCC type 2a lesions and one as a KCC type 1 lesion. The most extensive finding was a longitudinal erythematous mucosal erosion of 40 × 20 mm at 30 cm from the incisors (KCC type 2a) (Figure 4). Further findings were a small longitudinal ulceration of 6 mm in length surrounded by a slight erythema at 30 cm from the incisors (KCC type 2a), a circular erosion of 10 mm in diameter at 31 cm from the incisors (KCC type 2a), two reddish, partly fibrinous erosions of 20 mm at 25 cm from the incisors (KCC type 2a), a small erythematous lesion of 3 mm at 22 cm from the incisors (KCC type 1), and a small fissural lesion of 2 × 5 mm at 33 cm from the incisors (KCC type 2a). None of these patients developed clinical symptoms, such as odynophagia, hemoptysis or symptomatic reflux. Two patients denied further endoscopic follow-up. Healing was documented in three patients during a follow-up endoscopy within 2 weeks after the procedure and in one patient during a third endoscopy 5 weeks after the procedure (Figure 4). Notably, only 8% (*n* = 11) of the total cohort showed entirely unsuspicious endoscopy results. In 120 patients, gastritis (*n* = 104), glycogen acanthosis (*n* = 24), esophagitis (*n* = 23), hernia (*n* = 19), Barrett’s esophagus (*n* = 16), varices (*n* = 10) and other abnormalities (*n* = 24) were noticed.

The overall adverse event rate was 7% and included minor complications only. A complete list of adverse events is shown in Table 4. Complications occurred significantly more often in EDEL(+) patients (*p* < 0.01). In the EDEL(+) group, two cases of pericarditis accompanied by a small inflammatory pericardial effusion could be treated conservatively, and one mild PV stenosis (LIPV, asymptomatic) was incidentally noticed in a cardiac MRI that was performed for another indication. No symptoms of gastroparesis, no atrio-esophageal fistula and no MACCE occurred. No patient suffered from permanent sequelae.

### 3.2. Pre-Procedural Indices of EDEL

Patients with EDEL were less frequently overweight (1/6 [17%] vs. 85/125 [68%]; *p* = 0.02), tended to have a lower BMI (24.5 ± 4.1 vs. 27.4 ± 4.1; *p* = 0.09) and showed significantly higher creatinine levels (1.55 ± 1.18 vs. 1.07 ± 0.42 mg/dL; *p* = 0.02) as compared to EDEL(−) patients, respectively. All other parameters, including the CHA_2_DS_2_-VASc score, C-reactive protein and white blood cell count before or after the procedure, did not show significant differences between groups (Table 1).

### 3.3. Procedural Indices of EDEL

Procedural data are summarized in Table 2 and Table 3. There were neither significant differences in common procedural data, nor were there significant differences in the locally applied contact force (maximum, average), duration of RF-applications or the total energy in the four pre-defined regions of the posterior LA wall segments of the wide antral ablation line, e.g., left and right postero-superior and postero-inferior around the PV. Only the combined contact force indices, e.g., force-time-integral (2972 ± 3267 vs. 1757 ± 1262 g-s; *p* = 0.04) and force-power-time-integral (83,547 ± 105,940 vs. 43,556 ± 35,255 g●J; *p* = 0.02), demonstrated significantly higher values for the postero-inferior site of the left PV in EDEL(+) patients as compared to EDEL(−) patients. In contrast, at the postero-superior site, no statistically significant differences were noted.

## 4. Discussion

In this single-center prospective study, patients underwent contact force-guided RF-PVI without esophageal temperature monitoring. We evaluated the incidence of EDEL by means of post-procedural endoscopy and the contribution of applied contact force to EDEL formation. For the latter, four pre-defined ablation sites of the wide antral circumferential line that are located in close anatomic proximity to the esophagus were analyzed, e.g., the postero-superior and postero-inferior segment of the LA wall around the left- and right-sided PVs (Figure 2). Additionally, patient characteristics were compared and the clinical outcome was assessed. The key findings are: (1) The incidence of EDEL accounted for 4.6% under the limitation of energy, duration of RF-ablation and contact force; (2) Only the combined ablation indices (force-time-integral and force-power-time-integral) at the posterior-inferior site of the left PV were related to the occurrence of EDEL; (3) EDEL(+) patients were less frequently overweight and had higher creatinine levels; (4) at 12 months, freedom from arrhythmia was 74% in paroxysmal and persistent AF patients.

The incidence of esophageal thermal lesions exceeded 10% in the majority of previous publications [5,6,7]. General anesthesia, non-irrigated ablation catheters, high power output above 25–30 W, a long application duration beyond 20–30 s [6], the use of an 8 mm tip ablation catheter [25] and other factors have been described as risk factors for esophageal thermal lesions or atrio-esophageal fistulas. In our cohort, all ablation procedures were performed under conscious sedation using a 3.5 mm irrigated tip catheter and our ablation settings for the posterior LA wall were limited for energy (30 W), ablation duration (30 s) and contact force (40 g), which could have contributed to the comparably low incidence of EDEL. Apart from this, no other significant approaches to minimize esophageal complications such as esophageal cooling or deviation have been established in daily practice. The ablation index was not available in our center at the time of patient inclusion. However, the ablation index was developed by means of maximizing efficacy (i.e., high first pass isolation, shortening procedure time) and was not safety driven with respect to prevent esophageal lesions [26]. The ablation index results from an integrated formula that continuously adapts power, contact force, and time during RF ablation. A recent meta-analysis demonstrated increased efficacy without improving the safety profile compared to non-ablation index-guided catheter ablation [27] and others reported a comparable incidence of EDEL after ablation index-guided PVI [28]. Combined contact force indices that are also incorporated in the ablation index were of particular interest in this study and were therefore separately calculated.

### 4.1. Esophageal Temperature Monitoring

The net clinical benefit of esophageal temperature monitoring during PVI is still under debate and is only a class IIA/C recommendation in the guidelines [14]. Most recently, the randomized OPERA trial did not show advantages of esophageal temperature monitoring regarding the occurrence of EDEL in RF ablation [15]. Others reported the use of esophageal temperature monitoring per se as a risk factor for EDEL formation [29]. Additionally, multiple temperature probes with different technical features are available. However, reliable temperature detection is not always possible (longitudinal vs. S-shaped probes, single vs. multi-thermocouples, suboptimal probe placement, limited tissue contact, etc.) [30], which can raise safety concerns for the procedure and explain EDEL occurrence despite esophageal temperature monitoring. In this study, the prevalence of EDEL was low (4.6%) even without esophageal temperature monitoring, which contributes to the debate of whether or not temperature monitoring should be recommended within a standardized setting in every AF ablation procedure. The present results and earlier conflicting findings regarding the use of esophageal temperature monitoring implicate the importance of developing further strategies to prevent esophageal complications, e.g., by focusing on procedure- and patient-specific characteristics as performed in this study.

### 4.2. Procedural Indices of EDEL

Our data demonstrate that only the combined ablation indices, e.g., force-time-integral and force-power-time-integral, were significantly greater during ablation at the left posterior-inferior site of the left PV in EDEL(+) patients. Comparison of the other procedural data relating to the four investigated LA sites in patients with or without EDEL did not reveal significant differences with respect to contact force duration, applied energy and maximal or average contact force. Tsao, et al. [31] investigated the anatomical relationship of the esophagus and the LA, which are solely separated by a thin and discontinuous fibrofatty layer. They described one leading anatomic route (90% of subjects) of the esophagus passing alongside the LA posterior wall next to the ostia of the left PVs. Of all PVs, the distance to the esophagus was shortest from the left inferior PV (LIPV: 2.8 ± 2.5 mm, LSPV: 10.1 ± 3.4 mm, RIPV: 19.6 ± 7.0 mm, RSPV: 28.4 ± 6.1 mm), which strongly supports our findings and is well in line with the study of Piorkowski, et al. [32]. They investigated electroanatomical reconstructions and computed tomography of the LA and the esophagus, demonstrating a direct contact of the esophagus to the inferior region of the left PV in 99% (3D mapping system) and 97% (CT).

### 4.3. Patient Characteristics as Indices of EDEL

Furthermore, Tsao, et al. described the thinnest fat plane between the esophagus and LA in the region of the inferior PVs [31]. Others reported a positive correlation of a lower BMI with a shorter LA-esophagus distance [33], which is a result of smaller fat pads between the two structures and lesser or even no pericardial fat, which both serve as a thermal insulator during RF-energy delivery [34]. In our cohort, patients with EDEL were less frequently overweight and tended to have a lower BMI, which supports these observations. Notably, the CHA_2_DS_2_-VASc score, which also circumscribes the degree of morbidity, did not affect the outcome. More data are necessary to demonstrate if thinner patients or patients with impaired kidney function are more likely to experience EDEL after RF-ablation.

### 4.4. Adverse Event Rate apart from EDEL

Despite an overall low adverse event rate without any major complications, patients with EDEL experienced significantly more complications. Two patients with pericarditis and one patient with a mild, asymptomatic PV stenosis (LIPV, incidental finding) were observed in the EDEL(+) group. Hypothetically, a greater force-time-integral and/or force-power-time-integral that lead to EDEL may also induce pericarditis on the proximal side of the esophagus.

### 4.5. Clinical Implications and Future Perspectives

So far, esophageal temperature monitoring has experienced widespread use during PVI and is the standard of care in several centers, but no significant reduction of EDEL or atrio-esophageal fistulas has been demonstrated. Therefore, Kadado et al. concluded to reduce delivered energy and catheter contact force in the posterior left atrium [35]. The results of our study add important additional information that could be implemented in future strategies to prevent esophageal lesions. Firstly, the present findings question the necessity of esophageal temperature monitoring in every patient. We have shown that contact force-guided PVI with preset limits of power, ablation duration, and contact force for ablation at posterior segments of the antral line around the PVs can be safely and efficiently performed without esophageal temperature monitoring. Freedom from arrhythmia was 74% at 12 months, which is similar as compared to current literature for PVI [4]. Six patients (4.6%) experienced asymptomatic EDEL, and no atrio-esophageal fistula, MACCE or permanent sequelae occurred. Whether certain patient populations with risk factors for esophageal thermal lesions can benefit from esophageal temperature monitoring, e.g., lower body weight or higher creatinine levels in our study requires further investigation. The systematic use of esophageal temperature monitoring with special algorithms is valuable for scientific evaluations or new catheters but is not absolutely necessary for procedures with a standard approach. Secondly, a careful adaptation of the ablation settings with additional consideration of combined ablation indices (force-time-integral, force-power-time-integral) could improve safety and be implemented in contact force ablation algorithms. Furthermore, biophysics of tissue heating is a valuable factor to be considered during RF ablation. Recently, novel RF ablation strategies to perform PVI were introduced in clinical practice, e.g., (very) high power short duration (HPSD) ablation titrating power from 45–90 W. Compared to conventional RF ablation, the HPSD strategy leads to a shift of tissue heating from conductive to resistive heating with an improved more uniform transmural lesion geometry, but a decreased depth effect [36]. This could have a protective impact on surrounding structures, e.g., the esophagus and improve procedural success. Only recently, the incidence of EDEL was reported 6% after HPSD ablation [37] and 0% in very HPSD ablation [38].

## 5. Limitations

This study has several limitations. It is a single-center cohort study with prospective data analyses of a relatively small sample size. The ablation index was not available during the time of patient inclusion. Few subjects developed esophageal thermal lesions, which can affect the statistical power of the results and does not allow for conclusions regarding the incidence of atrio-esophageal fistulas.

## 6. Conclusions

Contact force-guided PVI with considerate settings for ablation at the posterior segments of the wide antral ablation line causes a low incidence of asymptomatic, self-limiting, endoscopically detected esophageal thermal lesions despite omitting esophageal temperature monitoring. Contact force-sensing technologies with incorporated indices, e.g., force-time-integral and force-power-time-integral, should be taken into account regarding optimized contact force algorithms to reduce esophageal lesion formation and enhance procedural safety. Future studies need to clarify whether esophageal temperature monitoring and/or different contact force strategies can benefit certain patient populations at increased risk for esophageal injury, e.g., patients with low body weight or elevated creatinine levels.

## Figures and Tables

**Table 1:** Baseline characteristics.

	Total *n* = 131	EDEL(−) *n* = 125	EDEL(+) *n* = 6	*p*-Value
Age (years)	65.5 ± 10.3	65.2 ± 10.3	71.2 ± 7.0	0.17
Male sex, *n* (%)	75 (57)	72 (58)	3 (50)	1.0
Body-Mass-Index (kg/m^2^)	27.3 ± 4.2	27.4 ± 4.1	24.5 ± 4.1	0.09
Overweight/Obesity, *n* (%)	86 (66)	85 (68)	1 (17)	0.02
- Overweight, *n* (%)	56 (43)	56 (45)	0 (0)	0.12
- Obesity Class I, *n* (%)	23 (18)	22 (18)	1 (17)	
- Obesity Class II, *n* (%)	3 (2)	3 (2.4)	0 (0)	
- Obesity Class III, *n* (%)	3 (2)	3 (2.4)	0 (0)	
Arterial hypertension, *n* (%)	92 (70)	87 (70)	5 (83)	0.67
Dyslipidemia, *n* (%)	57 (44)	55 (44)	2 (33)	0.70
Diabetes mellitus, *n* (%)	14 (11)	14 (11)	0 (0)	1.0
CAD, *n* (%)	40 (31)	37 (30)	3 (50)	0.37
PAD, *n* (%)	7 (5)	7 (6)	0 (0)	1.0
History of stroke, *n* (%)	5 (4)	4 (3)	1 (17)	0.21
Heart failure, *n* (%)	13 (10)	12 (10)	1 (17)	0.47
History of heart surgery, *n* (%)	8 (6)	8 (6)	0 (0)	1.0
COLD, *n* (%)	10 (8)	9 (7)	1 (17)	0.39
CKD, *n* (%)	34 (26)	31 (25)	3 (50)	0.18
NYHA class	1.7 ± 0.9	1.7 ± 0.9	2.2 ± 1.0	0.22
- NYHA I, *n* (%)	68 (52)	66 (53)	2 (33)	0.2
- NYHA II, *n* (%)	35 (27)	34 (27)	1 (17)	
- NYHA III, *n* (%)	23 (18)	20 (16)	3 (50)	
- NYHA IV, *n* (%)	5 (4)	5 (4)	0 (0)	
EHRA Score				
- EHRA IIa, *n* (%)	26 (20)	25 (20)	1 (17)	0.19
- EHRA IIb, *n* (%)	13 (10)	13 (10)	0 (0)	
- EHRA III, *n* (%)	80 (61)	77 (62)	3 (50)	
- EHRA IV, *n* (%)	12 (9)	10 (8)	2 (3)	
Paroxysmal AF, *n* (%)	75 (57)	70 (56)	5 (83)	0.41
Persistent AF, *n* (%)	51 (39)	50 (40)	1 (17)	
Atrial Tachycardia, *n* (%)	5 (4)	5 (4)	0 (0)	
CHA₂DS₂-VASc-Score, *n* (%)	2.5 ± 1.6	2.5 ± 1.6	3.3 ± 1.2	0.19
Ejection fraction (%)	60.2 ± 10.8	60.3 ± 10.4	57 ± 17.4	0.46
LA diameter (mm)	40.6 ± 7.5	40.9 ± 7.6	40.4 ± 6.1	0.89
PPI at baseline, *n* (%)	45 (34)	43 (34)	2 (33)	1.0
Anticoagulation, *n* (%)	130 (99)	124 (99)	6 (100)	1.0
- Phenprocoumon, *n* (%)	63 (48)	61 (49)	2 (33)	0.68
- Dabigatran, *n* (%)	8 (6)	8 (6.4)	0 (0)	1.0
- Apixaban, *n* (%)	18 (14)	17 (14)	1 (17)	1.0
- Rivaroxaban, *n* (%)	39 (30)	36 (29)	3 (50)	0.36
- Enoxaparin, *n* (%)	2 (2)	2 (1.6)	0 (0)	1.0
Creatinine (mg/dL)	1.09 ± 0.48	1.07 ± 0.42	1.55 ± 1.18	0.02
GFR (mL/min)	70.6 ± 19.6	71.3 ± 19.1	56.3 ± 26.6	0.07
C-reactive protein (mg/dL), initially	0.33 ± 0.44	0.34 ± 0.44	0.25 ± 0.19	0.63
Leukocytes (×1.000/µL), initially	7.2 ± 2.1	7.2 ± 2.1	8.4 ± 1.9	0.16
C-reactive protein (mg/dL), post abl.	1.92 ± 2.57	1.97 ± 2.62	0.92 ± 0.65	0.33
Leukocytes (×1.000/µL), post abl.	8.6 ± 2.7	8.6 ± 2.7	8.7 ± 2.9	0.98
Any endoscopic pathology ^a^, *n* (%)	120 (92)	116 (93)	4 (67)	0.13
- Esophagitis, *n* (%)	23 (18)	22 (18)	1 (17)	0.90

Data are presented as mean ± SD, or *n* (%) of patients. CAD indicates coronary artery disease; PAD, peripheral artery disease; COLD, chronic obstructive lung disease; CKD, chronic kidney disease; AF, atrial fibrillation; LA, left atrium; PPI, proton pump inhibitor; INR, international normalized ratio; and GFR, glomerular filtration rate. ^a^ other than EDEL.

**Table 2:** Procedural data.

	Total *n* = 131	EDEL(−) *n* = 125	EDEL(+) *n* = 6	*p*-Value
Total procedure time (min)	134 ± 30	133 ± 30	138 ± 23	0.73
Fluoroscopy time (min)	11.7 ± 5.6	11.5 ± 5.0	14.7 ± 8.5	0.18
Dose area product (cGy)	2214 ± 1519	2199 ± 1480	2879 ± 2159	0.28
Total duration of RF applications at posterior wall segments during WACA (s)	785 ± 323	781 ± 324	871 ± 336	0.51
Right-sided pulmonary veins	475 ± 223	478 ± 221	403 ± 277	0.93
Left-sided pulmonary veins	352 ± 132	350 ± 125	388 ± 240	0.50
Total energy at posterior wall segments during WACA (J)	19,223 ± 8129	19,065 ± 8023	22,514 ± 10,403	0.31
Average contact force at posterior wall segments during WACA (g)	16.8 ± 3.7	16.1 ± 3.8	16.8 ± 3.7	0.66

Data are presented as mean ± SD. Table summarizes procedural data of contact force-guided RF ablation with respect to the entire cohort and patients with or without EDEL, respectively. WACA indicates wide antral circumferential ablation.

**Table 3:** Procedural data related to the posterior segments of the wide antral circumferential ablation line around the pulmonary veins.

	EDEL(−) *n* = 125	EDEL(+) *n* = 6	*p*-Value
Total energy of RF appl. per patient (J)			
RSPV, postero-superior	7147 ± 4027	6407 ± 1763	0.66
RIPV, postero-inferior	4637 ± 2417	6039 ± 2130	0.17
LSPV, postero-superior	5238 ± 2381	5661 ± 4154	0.67
LIPV, postero-inferior	3357 ± 1961	4407 ± 3227	0.22
Average CF during RF application (g)			
RSPV, postero-superior	18.3 ± 5.0	19.6 ± 5.9	0.55
RIPV, postero-inferior	15.2 ± 5.1	15.8 ± 6.1	0.80
LSPV, postero-superior	17.4 ± 5.9	20.4 ± 4.3	0.23
LIPV, postero-inferior	12.6 ± 4.2	14.6 ± 6.8	0.26
Force-time-integral (g·s)			
RSPV, postero-superior	5142 ± 2790	4799 ± 1241	0.77
RIPV, postero-inferior	2764 ± 1507	3432 ± 1208	0.80
LSPV, postero-superior	3531 ± 1663	4337 ± 2897	0.27
LIPV, postero-inferior	1757 ± 1262	2972 ± 3267	0.04
Force-power-time-integral (g·J)			
RSPV, postero-superior	127,604 ± 69,257	119,415 ± 28,710	0.77
RIPV, postero-inferior	69,401 ± 39,511	89,623 ± 31,000	0.22
LSPV, postero-superior	88,129 ± 41,716	114,215 ± 84,599	0.16
LIPV, postero-inferior	43,556 ± 35,255	83,547 ± 105,940	0.02

Data are presented as mean ± SD. Table shows different parameters of contact force RF-PVI at the postero-superior and postero-inferior segments of the wide antral ablation line around the left and right pulmonary veins. LSPV indicates left superior pulmonary vein; LIPV, left inferior PV; RSPV, right superior PV; and RIPV, right inferior PV.

**Table 4:** Adverse event rate.

	EDEL(−) *n* = 125	EDEL(+) *n* = 6	*p*-Value
Major complications, *n* (%)	0 (0)	0 (0)	1.0
Pericardial tamponade, *n* (%)	0 (0)	0 (0)	1.0
Gastroparesis, *n* (%)	0 (0)	0 (0)	1.0
Atrioesophagela fistula, *n* (%)	0 (0)	0 (0)	1.0
TIA, *n* (%)	0 (0)	0 (0)	1.0
Stroke/MI/death, *n* (%)	0 (0)	0 (0)	1.0
Minor complications, *n* (%)	6 (5)	3 (50)	<0.01
Pericarditis, *n* (%)	0 (0)	2 (33)	<0.01
Mild PV stenosis ^a^, *n* (%)	0 (0)	1 (17)	0.03
Groin complications, *n* (%)	4 (3)	0 (0)	0.65
- AV fistula, *n* (%)	1 (1)	0 (0)	0.82
- Hematoma, *n* (%)	3 (2)	0 (0)	0.70
Pulmonary infection, *n* (%)	2 (2)	0 (0)	0.76
Overall complications, *n* (%)	9 (7)		

Data are presented as numbers and percentages. Table shows the adverse event rate (EDEL excluded) of patients with or without EDEL.^a^ One mild PV stenosis (<50% lumen) occurred in the left inferior pulmonary vein (incidental finding, asymptomatic). TIA indicates transient ischemic attack; MI, myocardial infarction; AV, arteriovenous and PV, pulmonary vein.

## Data Availability

Raw data were generated at the University Hospital in Düsseldorf, Germany. The data that support the findings of this study are available from the corresponding author on reasonable request.
